# Aging reprograms microglia toward an inflammasome-linked response to traumatic brain injury

**DOI:** 10.1172/JCI207022

**Published:** 2026-06-15

**Authors:** Josh M. Morganti, Adam D. Bachstetter

**Affiliations:** 1 Department of Neuroscience,; 2 Spinal Cord and Brain Injury Research Center, and; 3 Sanders-Brown Center on Aging, University of Kentucky, Lexington, Kentucky, USA.

## Abstract

Traumatic brain injury (TBI) disproportionately kills and disables older adults, yet the biology driving this vulnerability remains unresolved. In this issue of the *JCI*, Lu et al. combined single-cell transcriptomics, metabolomics, and chromatin profiling in mice, validated in human TBI tissue, to define an age-dependent microglial dichotomy. They report that an NLRP3^+^/IL-1β–linked state dominates the aged brain, while a Lysozyme^+^/Lyz2^+^ state predominates in the young. Microglia-targeted perturbation of NLRP3 and ELF1 each shifted the balance and improved survival in mouse models of TBI, and the repurposed drug Imeglimin improved outcomes in these models, confirming that this pathway is druggable. By connecting NLRP3 inflammasome dominance, ELF1-driven transcription, and glycolytic reprogramming to the loss of a protective Lyz2^+^ response, this work converts age from a clinical risk factor to a set of druggable microglial targets.

## Age reshapes the injury response

Older adults who experience traumatic brain injury (TBI) sustain the highest hospital admission rates of all age groups and face mortality rates more than double that of younger patients; moreover, among survivors, older adults endure prolonged time to resolution of functional dependence outcomes (1). These figures reflect more than frailty and comorbidities in the aged population, as the aged brain itself mounts a more damaging secondary injury response. Even without injury, the aged brain has a distinct microglial transcriptional program (2) and lipid droplet–accumulating microglia that are phagocytosis impaired, ROS high, and hyperresponsive to inflammatory challenge (3), all within a white matter environment that is burdened by progressive myelin degeneration (4). When the aged brain suffers a TBI, these changes compound to produce impaired microglial phagocytosis, elevated IL-1β, NOX2-linked oxidative stress, and later a persistent disease-associated microglia–like (DAM-like) inflammatory program with white matter loss, alongside exaggerated CCR2^+^ myeloid recruitment that can be pharmacologically targeted to improve recovery (5–7). The aged inflammatory response to a TBI is also shaped by age-sensitive changes in astrocytes (8), meninges, spleen, and white matter (9, 10).

## Aging shifts microglial state after TBI

**Figure 1:**
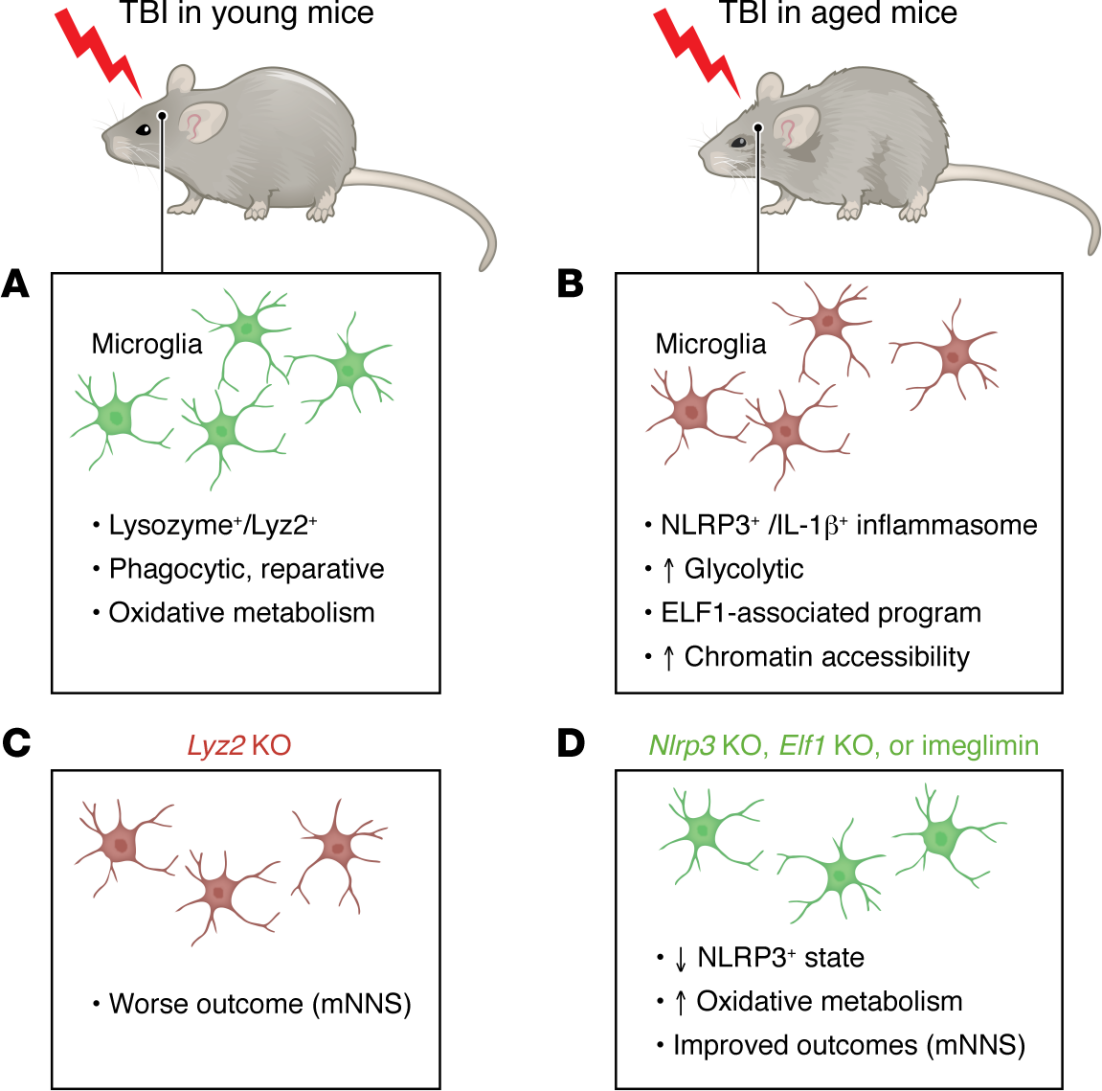
Aging redirects the post-TBI microglial response toward an inflammasome-linked state with distinct metabolic, chromatin, and transcriptional features. Lu et al. (11) used a model of TBI and compared outcomes in young and aged mice. Microglial responses to injury diverged by age, with young adult mice preferentially mounting a Lysozyme/Lyz2^+^ response and aged adult mice preferentially developing an NLRP3^+^/IL-1β^+^ state. (**A**) In young injured brains, microglia were enriched for a Lysozyme/Lyz2^+^ state, characterized by lysosomal and phagocytic gene programs (*Lyz2*, *Cst7*, *Spp1*), partial concordance with DAM, and reliance on oxidative metabolism. (**B**) In aged injured brains, microglia were enriched for an NLRP3^+^/IL-1β^+^ inflammasome-linked state marked by inflammatory genes (*Nlrp3*, *Il1b*, *Casp1*), a glycolytic shift, increased chromatin accessibility at inflammatory and senescence-associated loci (*Nlrp3*, *Casp1*, *Il1b*, *Il18*, *Cdkn1a*, *Cdkn2a*), and an ELF1-associated transcriptional program. (**C**) In young mice, microglial *Lyz2* deletion worsened neurological outcome following TBI, as measured by the modified Neurological Severity Score (mNNS), supporting a beneficial or at least nonharmful role for the Lysozyme^+^/Lyz2^+^ state. (**D**) In aged mice, microglial Nlrp3 deletion, microglia-selective *Elf1* ablation, or treatment with the oxidative phosphorylation inhibitor Imeglimin each shifted the response away from the NLRP3^+^ state and improved outcome. Moreover, ELF1 loss and Imeglimin also normalized metabolic readouts in microglial cell systems. Together, these findings support a model in which aging redirects the post-TBI microglial response away from a reparative Lysozyme^+^/Lyz2^+^ program and toward a maladaptive inflammasome-linked state.

In this issue of the *JCI*, Lu et al. performed controlled cortical impact in aged (16–18 months) and young (6–8 weeks) mice and profiled the injured hemisphere at 72 hours by scRNA-seq (11) (Figure 1). Among 12 cell types, microglia showed the largest age-associated transcriptional remodeling. Five microglial states emerged, but two carried the strongest association with age and outcome. A Lyz2^+^ cluster (*Lyz2*, *Cst7*, *Spp1*), with partial concordance to DAM markers and IL-4 responsiveness, predominated in young brains (Figure 1A). An NLRP3^+^ cluster (*Nlrp3*, *Il1b*, *Casp1*) was enriched in aged brains (Figure 1B). These findings provide cellular context to prior bulk-level observations of elevated IL-1β, NOX2-linked ROS, and senescence markers in aged TBI mice (6, 7). Flow cytometry, immunofluorescence, and a repetitive mild TBI model confirmed the shift (notably, both sexes were examined, with similar findings across the mouse experiments), and human surgical tissue from 13 young (<60 years) and 22 aged (>65 years) patients with TBI showed the same pattern, with NLRP3^+^ microglia enriched with aged patients and Lysozyme^+^ enriched in young patients.

The study then used genetic and pharmacological experiments to test the functional importance of these microglial states. Microglial *Lyz2* knockout worsened outcome in both age groups (Figure 1C), supporting the idea that aging involves not only greater inflammation, but also loss of a protective microglial population. Conversely, microglia-targeted *Nlrp3* knockout in aged mice reduced mortality and improved recovery (Figure 1D). MCC950, a selective NLRP3 inhibitor, reproduced this effect pharmacologically and also reduced brain lymphocyte infiltration, suggesting that the NLRP3^+^ state contributes to immune recruitment. Together, these findings indicate that the aged brain does not simply carry more inflammation, but a different inflammatory composition in which the balance between these two states is linked to outcome.

## Metabolic rewiring drives aged microglial inflammation

The two states were metabolically separable at every level tested (11). Seahorse assays showed elevated glycolysis with suppressed oxidative phosphorylation in NLRP3^+^ microglia. Untargeted metabolomics of FACS-purified microglia separated NLRP3^+^ and Lysozyme^+^ populations by principal component analysis, with NLRP3^+^ microglia showing reduced NADH/FADH2 equivalents and lower abundance of most measured TCA intermediates. Glucose isotope tracing confirmed increased glycolytic carbon flux, showing that while proinflammatory myeloid cells characteristically favor glycolysis, this divergence is not merely activation associated. It is age-exacerbated, consistent with the interpretation that aging locks microglia into a glycolytic program that stabilizes the inflammatory state. Whether this reflects coordinated immunometabolic reprogramming or underlying mitochondrial dysfunction that forces glycolytic dependence is unresolved. Aged microglia accumulate mitochondrial damage, and mitochondrial ROS, oxidized mtDNA, and impaired mitophagy are established NLRP3 activators. NOX2-derived ROS may further amplify this axis (12). Lu et al.’s findings highlight that mitochondrial stress and inflammatory commitment reinforce each other. NLRP3^+^ microglia showed increased accessibility at *Nlrp3*, *Casp1*, *Il1b*, and *Il18* as well as the senescence-associated genes *Cdkn1a* and *Cdkn2a* (11). This fits the microglial priming framework, in which aging leaves microglia epigenetically poised so that injury triggers an amplified and qualitatively different inflammatory response (13). The *Cdkn1a*/*Cdkn2a* accessibility is notable but should not be overinterpreted as canonical senescence at the 72-hour time point.

## ELF1 organizes the microglial NLRP3^+^ program

To investigate the factors underlying NLRP3^+^ microglia formation, Lu et al. combined the SCENIC algorithm with cross-species transcription factor filtering, ATAC-seq motif enrichment, and CRISPR screening (11). This approach prioritized ELF1 from a list of eight candidates determined to significantly promote NLRP3^+^ microglia formation. ELF1 is not typically viewed as a core inflammatory transcription factor. ETS family members ELF1 and PU.1 activate the *CYBB*/gp91phox promoter in phagocytes (14), linking it to NOX2-mediated ROS generation. Additionally, ELF1 controls an innate immunity transcriptional program required for rapid LPS-responsive gene expression (15). In Lu et al.’s analyses, ELF1 ablation had the strongest inhibitory effect among the transcription factor candidates screened, and three independent sgRNAs reproduced the reduction in NLRP3^+^ microglia formation (11). That ELF1 outperformed selected NF-κB– and AP-1–related candidates makes it a distinctive regulatory lead, although the precise level at which it acts within this program remains unresolved. Conditional *Elf1* ablation in *Cx3cr1*^c*reERT*2^ mice shifted microglia from NLRP3^+^ toward Lyz2^+^ identity, upregulated antiinflammatory genes (*Arg1*, *Tgfb1*, *Lyz2*, and *Sall1*), and improved survival in aged TBI mice (11). ELF1 ablation reduced the NLRP3^+^ state but did not eliminate it, which is consistent with ELF1 acting as a submodule of the inflammatory program rather than acting as a master switch. No outside evidence links ELF1 to direct regulation of core inflammasome components, and the *CYBB* promoter link (14) remains the strongest between ELF1 and broader inflammatory redox biology.

## Imeglimin repurposed to link transcription and metabolism

Lu et al. screened 8,561 compounds by virtual docking for potential ELF1 inhibitors, narrowed the list to 10 candidates with favorable blood-brain barrier properties, and found that Imeglimin, marketed as a antidiabetic agent, showed the strongest inhibition of NLRP3^+^ microglia among those shortlisted compounds (11). In their study, Imeglimin reduced NLRP3^+^ microglia, improved extracellular acidification rate/oxygen consumption rate (ECAR/OCR) readouts in microglial cell line experiments, increased survival, and lowered cerebrospinal fluid levels of IL-1β and IL-6 in TBI mice (11). In ELF1-knockout cells, Imeglimin produced no further reduction in NLRP3^+^ microglia and no additional improvement in ECAR/OCR, and after conditional knockout of *Elf1* in mice, it did not further reduce the brain NLRP3^+^ population (11). Outside this paper, Imeglimin’s most consistent biology is mitochondrial and metabolic remodeling, with narrower but real overlap with NLRP3/IL-1β attenuation in myeloid and microglia-like systems (16, 17). These data make Imeglimin most useful here as a mechanistically informative metabolic probe rather than a near-term clinical candidate for TBI, especially given that the limited support for its use in CNS conditions outside TBI comes mainly from ischemia models such as rat stroke (18).

## Limitations and future directions

The human data are a strength of Lu et al.’s study, but because the cohort included only 13 young and 22 aged TBI patients and lacked age-matched non-TBI controls, it is not possible to fully separate TBI-induced microglial changes from baseline aging effects. Moreover, the human arm remains limited to flow cytometry, representative immunofluorescence, and targeted RT-qPCR and therefore does not validate the full microglial state architecture, metabolic program, or functional role of ELF1 in human brain tissue. That said, the broader literature supports the translational relevance of the IL-1 pathway. Recombinant IL-1 receptor antagonist has been safely administered in human severe TBI (19), and IL-1R1 signaling mediates neuroinflammation and cognitive decline after experimental diffuse TBI (20).

All molecular profiling was performed at 72 hours, presenting a single snapshot of a dynamic process. Whether NLRP3^+^ dominance persists, resolves, or transforms in later phases is unknown. The *Lyz2* deletion data demonstrate the functional importance of this state, and the partial DAM concordance, phagocytic gene expression (*Cst7*, *Spp1*), and IL-4 responsiveness of these microglia suggest a reparative orientation, but whether the protective mechanism is primarily phagocytic, trophic, or antiinflammatory has not been determined. The study focuses on cell-intrinsic microglial programs but leaves the aged tissue milieu largely unaddressed. Aging increases brain myelin debris, lipid accumulation, and iron deposition, and these environmental inputs may shape the microglial state as powerfully as intrinsic programs (3, 4). The mechanistic chain from glycolysis through chromatin to ELF1 to the NLRP3^+^ state is presented as a connected model, but the pathway has been tested in parts rather than as a whole, and additional experiments will be needed to define causal direction.

Lu et al. provide one of the most integrative preclinical studies to date linking TBI outcome in aged individuals to acute microglial state composition. For a field that already knew aging worsens recovery through immune dysfunction, the advance here is specificity: the NLRP3/Lyz2 axis gives a concrete cellular framework, the ELF1 and metabolic data point to testable nodes, and the Imeglimin results show that the pathway is pharmacologically accessible. What remains is to determine whether these relationships hold over longer time courses and, ultimately, in human tissue.

## Conflict of interest

The authors have declared that no conflict of interest exists.

## Funding support

This work is the result of NIH funding, in whole or in part, and is subject to the NIH Public Access Policy. Through acceptance of this federal funding, the NIH has been given a right to make the work publicly available in PubMed Central.

- NIH grants R01NS120882, R01NS119165, R01AG070830, and R21NS147149.
- The Kentucky Spinal Cord and Head Injury Research Trust.
- Department of Defense grant AZ240194.

## Version 1 06/15/2026

Electronic publication

## References

[1] Ghneim M (2022). Traumatic brain injury in older adults: characteristics, outcomes, and considerations. Results from the American Association for the Surgery of Trauma Geriatric Traumatic Brain Injury (GERI-TBI) multicenter trial. J Am Med Dir Assoc.

[2] Olah M (2018). A transcriptomic atlas of aged human microglia. Nat Commun.

[3] Marschallinger J (2020). Lipid-droplet-accumulating microglia represent a dysfunctional and proinflammatory state in the aging brain. Nat Neurosci.

[4] Safaiyan S (2021). White matter aging drives microglial diversity. Neuron.

[5] Morganti JM (2016). Age exacerbates the CCR2/5-mediated neuroinflammatory response to traumatic brain injury. J Neuroinflammation.

[6] Ritzel RM (2019). Old age increases microglial senescence, exacerbates secondary neuroinflammation, and worsens neurological outcomes after acute traumatic brain injury in mice. Neurobiol Aging.

[7] Ritzel RM (2022). Functional and transcriptional profiling of microglial activation during the chronic phase of TBI identifies an age-related driver of poor outcome in old mice. Geroscience.

[8] Early AN (2020). Effects of advanced age upon astrocyte-specific responses to acute traumatic brain injury in mice. J Neuroinflammation.

[9] Macheda T (2024). Old age exacerbates white matter neuroinflammation and cognitive deficits following closed-head injury, particularly in female mice. Neurotrauma Rep.

[10] Moro F (2022). Ageing is associated with maladaptive immune response and worse outcome after traumatic brain injury. Brain Commun.

[11] Lu Z (2026). Aging-dependent microglial heterogeneity worsens outcomes in models of traumatic brain injury. J Clin Invest.

[12] Laabei J (2025). The NOX2-ROS-NLRP3 inflammasome axis in traumatic brain injury. J Neuroinflammation.

[13] Bray CE (2022). Chronic cortical inflammation, cognitive impairment, and immune reactivity associated with diffuse brain injury are ameliorated by forced turnover of microglia. J Neurosci.

[14] Voo KS, Skalnik DG (1999). Elf-1 and PU.1 induce expression of gp91(phox) via a promoter element mutated in a subset of chronic granulomatous disease patients. Blood.

[15] Varghese P (2025). The myeloid transcription factor Elf1 regulates genes with function in innate immunity. Exp Hematol.

[16] Kato H (2024). Imeglimin exhibits novel anti-inflammatory effects on high-glucose-stimulated mouse microglia through ULK1-mediated suppression of the TXNIP-NLRP3 axis. Cells.

[17] Lee JY (2024). Imeglimin attenuates NLRP3 inflammasome activation by restoring mitochondrial functions in macrophages. J Pharmacol Sci.

[18] Zemgulyte G (2022). Imeglimin is neuroprotective against ischemic brain injury in rats-a study evaluating neuroinflammation and mitochondrial functions. Mol Neurobiol.

[19] Helmy A (2014). Recombinant human interleukin-1 receptor antagonist in severe traumatic brain injury: a phase II randomized control trial. J Cereb Blood Flow Metab.

[20] Davis AC (2026). Interleukin-1 receptor-1 signaling mediates neuroinflammation, neuronal injury, and cognitive decline after diffuse traumatic brain injury. Brain Behav Immun.

